# 11-Hy­droxy-9-[1-(4-methyl­phen­yl)-4-oxo-3-phenyl­azetidin-2-yl]-18-oxo-10-oxa-2-aza­penta­cyclo­[9.7.0.0^1,8^.0^2,6^.0^12,17^]octa­deca-12,14,16-triene-8-carbonitrile

**DOI:** 10.1107/S1600536812044479

**Published:** 2012-11-07

**Authors:** Sivasubramanian Suhitha, Thothadri Srinivasan, Raju Rajesh, Raghavachary Raghunathan, Devadasan Velmurugan

**Affiliations:** a Centre of Advanced Study in Crystallography and Biophysics, University of Madras, Guindy Campus, Chennai 600 025, India; b Department of Organic Chemistry, University of Madras, Guindy Campus, Chennai 600 025, India

## Abstract

In the title compound, C_33_H_29_N_3_O_5_, the four-membered ring of the β-lactam fragment is essentially planar (r.m.s. deviation = 0.0122 Å), with the carbonyl O atom displaced from this ring by 0.856 (9) Å. The mean planes of the meth­oxy­phenyl and phenyl rings are inclined at dihedral angles 85.10 (7) and 21.56 (14)°, respectively, with respect to the mean plane of the four-membered ring. The pyrrolidine rings adopt envelope conformations with C atoms lying 0.535 (4) and 0.519 (4) Å out of the planes formed by the remaining ring atoms. The furan ring also adopts an envelope conformation with a C atom 0.560 (3) Å out of the plane formed by the remaining ring atoms. The nine-membered indene ring is almost planar (r.m.s. deviation = 0.0240 Å), with the carbonyl O atom displaced by 0.145 (3) Å from this ring. The mol­ecular structure is stabilized by a strong intra­molecular O—H⋯N hydrogen bond and the crystal structure is consolidated by C—H⋯O hydrogen bonds.

## Related literature

For general background to β-lactams, see: Brakhage (1998 ▶). For a related structure, see: Sundaramoorthy *et al.* (2012 ▶).
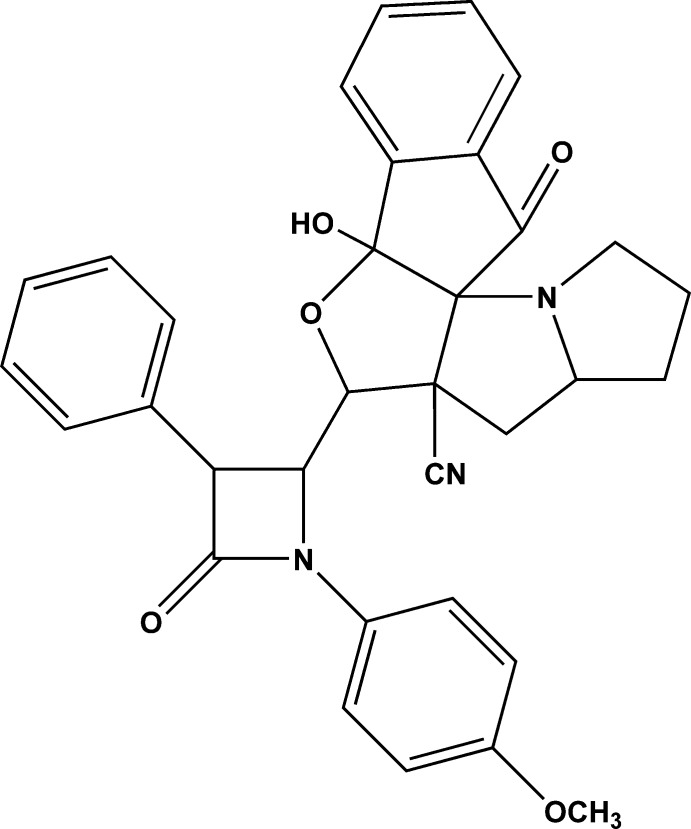


## Experimental

### 

#### Crystal data

C_33_H_29_N_3_O_5_
*M* *_r_* = 547.59
Orthorhombic, 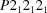

*a* = 10.2874 (16) Å
*b* = 14.138 (3) Å
*c* = 18.866 (3) Å
*V* = 2743.9 (8) Å^3^
*Z* = 4
Mo *K*α radiation
μ = 0.09 mm^−1^
*T* = 293 K
0.30 × 0.25 × 0.20 mm

#### Data collection

Bruker SMART APEXII area-detector diffractometer
Absorption correction: multi-scan (*SADABS*; Bruker, 2008 ▶) *T*_min_ = 0.973, *T*_max_ = 0.982
14810 measured reflections
6406 independent reflections
4551 reflections with *I* > 2σ(*I*)
*R*_int_ = 0.034

#### Refinement

*R*[*F* ^2^ > 2σ(*F* ^2^)] = 0.045
*wR*(*F* ^2^) = 0.123
*S* = 1.00
6406 reflections
375 parameters
H atoms treated by a mixture of independent and constrained refinement
Δρ_max_ = 0.25 e Å^−3^
Δρ_min_ = −0.16 e Å^−3^

### 

Data collection: *APEX2* (Bruker, 2008 ▶); cell refinement: *SAINT* (Bruker, 2008 ▶); data reduction: *SAINT*; program(s) used to solve structure: *SHELXS97* (Sheldrick, 2008 ▶); program(s) used to refine structure: *SHELXL97* (Sheldrick, 2008 ▶); molecular graphics: *ORTEP-3* (Farrugia, 1997 ▶); software used to prepare material for publication: *SHELXL97* and *PLATON* (Spek, 2009 ▶).

## Supplementary Material

Crystal structure: contains datablock(s) global, I. DOI: 10.1107/S1600536812044479/pv2596sup1.cif

Structure factors: contains datablock(s) I. DOI: 10.1107/S1600536812044479/pv2596Isup2.hkl

Supplementary material file. DOI: 10.1107/S1600536812044479/pv2596Isup3.cml

Additional supplementary materials:  crystallographic information; 3D view; checkCIF report

## Figures and Tables

**Table 1:** Hydrogen-bond geometry (Å, °)

*D*—H⋯*A*	*D*—H	H⋯*A*	*D*⋯*A*	*D*—H⋯*A*
C10—H10⋯O2^i^	0.98	2.43	3.337 (3)	154
C13—H13*A*⋯O2^i^	0.97	2.41	3.321 (3)	156
C29—H29⋯O4^i^	0.93	2.55	3.228 (3)	130
O4—H4*A*⋯N2	0.93 (3)	1.84 (3)	2.602 (3)	137 (3)

Symmetry code: (i) 
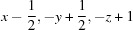
.

**Fig. 1.**
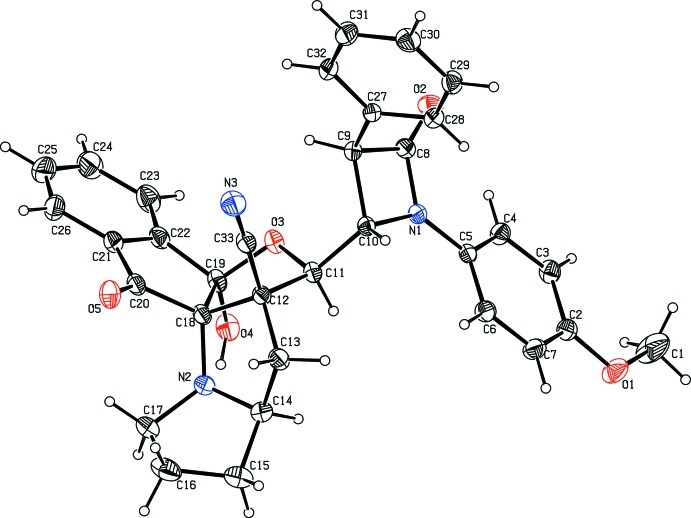
The molecular structure of the title compound, showing displacement ellipsoids drawn at the 30% probability level. H atoms are presented as a small spheres of arbitrary radius.

**Fig. 2.**
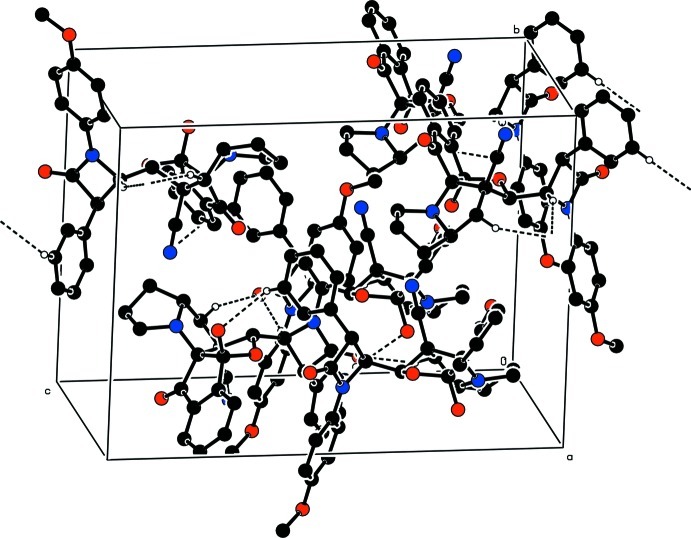
The crystal packing of the title compound viewed down a axis. H-atoms not involved in H-bonds have been excluded for clarity.

## supplementary crystallographic information

### Comment

The most commonly used β-lactam antibiotics for the therapy of infectious diseases are penicillin and cephalosporin (Brakhage, 1998). In view of potential applications, the crystal structure determination of the titled β-lactam derivative was carried out that is reported in this artiucle.

In the title compound (Fig. 1), the four membered ring of the β-lactam fragment (N1/C8–C10) is essentially planar (rmsd = 0.0122 Å) with O1 displaced from this ring by 0.856 (9) Å. The mean-planes of the benzene rings C2–C7 and C27–C32 are inclined at dihedral angles 85.10 (7) and 21.56 (14)°, respectively, with respect to the mean-plane of the four membered ring (N1/C8–C10).

The pyrrolidine rings (N1/C14–C17) and (N2/C12–C14/C18) adopt C16- and C13-envelope conformations with C16 and C13 atoms lying 0.535 (4) and 0.519 (4) Å, respectively, out of the planes formed by the remaining ring atoms. The furan ring (O3/C11/C12/C18/C19) also adopts a C11-envelope conformation with C11 atom 0.560 (3) Å out of the plane formed by the remaining ring atoms. The nine membered indene ring (C18–C26) is almost planar (rmsd = 0.0240 Å) with O5 displaced by 0.145 (3)Å from this ring.

The molecular structure of the title compound is stabilized by a strong intramolecular hydrogen bond O4—H4A···N2 (Table 1). The crystal structure is consolidated by intermolecular C—H···O hydrogen bonds (Tab. 1 & Fig. 2).

### Experimental

A reaction mixture of 2-(hydroxy(1-(4-methoxyphenyl)-4-oxo-3-phenylazetidin -2-yl)methyl)acrylonitrile (1.0 mmol), ninhydrine (1.1 mmol) and proline (1.1 mmol) was refluxed in methanol (20 ml) until completion of the reaction was evidenced by TLC analysis. After completion of the reaction the solvent was evaporated under reduced pressure. The crude reaction mixture was dissolved in dichloromethane (2 x 50 ml) and washed with water followed by brine solution. The organic layer was separated and dried over sodium sulfate. After filteration and evaporation of the organic solvent was carried out under reduced pressure. The product was separated by column chromatography using hexane and ethyl acetate (4:6) as an eluent to give a colorless solid. The product was dissolved in chloroform (3 ml) and heated for two minutes. The resulting solution was subjected to crystallization by slow evaporation of the solvent resulting in single crystals suitable for X-ray crystallographic studies.

### Refinement

All H atoms bonded to C-atoms were positioned geometrically and refined using a riding model, with C—H = 0.93, 0.96, 0.97 and 0.98 Å, for aryl, methyl, methylene and methine H-atoms, respectively. The *U*_iso_(H) were allowed at 1.5*U*_eq_(C methyl) or 1.2*U*_eq_(C non-methyl). The hydroxy H-atom was located from a difference map and was alloewed to refine freely. An absolute structure was not established due to insufficient anomalous dispersion effects. Therefore, 2699 Friedel pairs of reflections were merged.

### Crystal data

**Table:**

C_33_H_29_N_3_O_5_	*F*(000) = 1152
*M**_r_* = 547.59	*D*_x_ = 1.326 Mg m^−^^3^
Orthorhombic, *P*2_1_2_1_2_1_	Mo *K*α radiation, λ = 0.71073 Å
Hall symbol: P 2ac 2ab	Cell parameters from 6406 reflections
*a* = 10.2874 (16) Å	θ = 1.8–28.0°
*b* = 14.138 (3) Å	µ = 0.09 mm^−^^1^
*c* = 18.866 (3) Å	*T* = 293 K
*V* = 2743.9 (8) Å^3^	Block, colourless
*Z* = 4	0.30 × 0.25 × 0.20 mm

### Data collection

**Table:**

Bruker SMART APEXII area-detector diffractometer	6406 independent reflections
Radiation source: fine-focus sealed tube	4551 reflections with *I* > 2σ(*I*)
Graphite monochromator	*R*_int_ = 0.034
ω and φ scans	θ_max_ = 28.0°, θ_min_ = 1.8°
Absorption correction: multi-scan (*SADABS*; Bruker, 2008)	*h* = −7→13
*T*_min_ = 0.973, *T*_max_ = 0.982	*k* = −18→18
14810 measured reflections	*l* = −24→23

### Refinement

**Table:**

Refinement on *F*^2^	Primary atom site location: structure-invariant direct methods
Least-squares matrix: full	Secondary atom site location: difference Fourier map
*R*[*F*^2^ > 2σ(*F*^2^)] = 0.045	Hydrogen site location: inferred from neighbouring sites
*wR*(*F*^2^) = 0.123	H atoms treated by a mixture of independent and constrained refinement
*S* = 1.00	*w* = 1/[σ^2^(*F*_o_^2^) + (0.0605*P*)^2^ + 0.2909*P*] where *P* = (*F*_o_^2^ + 2*F*_c_^2^)/3
6406 reflections	(Δ/σ)_max_ < 0.001
375 parameters	Δρ_max_ = 0.25 e Å^−^^3^
0 restraints	Δρ_min_ = −0.16 e Å^−^^3^

### Special details

**Table:**

Geometry. All esds (except the esd in the dihedral angle between two l.s. planes) are estimated using the full covariance matrix. The cell esds are taken into account individually in the estimation of esds in distances, angles and torsion angles; correlations between esds in cell parameters are only used when they are defined by crystal symmetry. An approximate (isotropic) treatment of cell esds is used for estimating esds involving l.s. planes.
Refinement. Refinement of F^2^ against ALL reflections. The weighted R-factor wR and goodness of fit S are based on F^2^, conventional R-factors R are based on F, with F set to zero for negative F^2^. The threshold expression of F^2^ > 2sigma(F^2^) is used only for calculating R-factors(gt) etc. and is not relevant to the choice of reflections for refinement. R-factors based on F^2^ are statistically about twice as large as those based on F, and R- factors based on ALL data will be even larger.

### Fractional atomic coordinates and isotropic or equivalent isotropic displacement parameters (Å^2^)

**Table:**

	*x*	*y*	*z*	*U*_iso_*/*U*_eq_	
C1	0.5616 (4)	0.7130 (2)	0.3845 (2)	0.1143 (16)	
H1A	0.6495	0.7115	0.4016	0.171*	
H1B	0.5356	0.7775	0.3770	0.171*	
H1C	0.5562	0.6789	0.3407	0.171*	
C2	0.4988 (2)	0.57555 (16)	0.45053 (11)	0.0485 (6)	
C3	0.6029 (2)	0.52399 (16)	0.42370 (12)	0.0491 (5)	
H3	0.6624	0.5527	0.3935	0.059*	
C4	0.6177 (2)	0.42957 (16)	0.44211 (12)	0.0456 (5)	
H4	0.6874	0.3951	0.4243	0.055*	
C5	0.5287 (2)	0.38631 (14)	0.48714 (9)	0.0364 (4)	
C6	0.4241 (2)	0.43760 (16)	0.51336 (11)	0.0461 (5)	
H6	0.3636	0.4087	0.5429	0.055*	
C7	0.4105 (3)	0.53205 (17)	0.49536 (13)	0.0538 (6)	
H7	0.3412	0.5667	0.5136	0.065*	
C8	0.6046 (2)	0.21567 (16)	0.47055 (11)	0.0394 (5)	
C9	0.5463 (2)	0.14150 (15)	0.52086 (10)	0.0386 (5)	
H9	0.6120	0.1086	0.5493	0.046*	
C10	0.4835 (2)	0.22795 (14)	0.56050 (10)	0.0363 (4)	
H10	0.3884	0.2277	0.5571	0.044*	
C11	0.5303 (2)	0.25097 (16)	0.63567 (10)	0.0394 (5)	
H11	0.5225	0.3194	0.6429	0.047*	
C12	0.4597 (2)	0.19991 (15)	0.69836 (10)	0.0384 (5)	
C13	0.3429 (2)	0.25415 (18)	0.72944 (12)	0.0486 (6)	
H13A	0.2913	0.2827	0.6921	0.058*	
H13B	0.2880	0.2125	0.7572	0.058*	
C14	0.4057 (2)	0.32998 (17)	0.77630 (12)	0.0510 (6)	
H14	0.4282	0.3856	0.7478	0.061*	
C15	0.3287 (3)	0.3600 (2)	0.84225 (15)	0.0708 (8)	
H15A	0.3489	0.4247	0.8553	0.085*	
H15B	0.2359	0.3547	0.8340	0.085*	
C16	0.3722 (4)	0.2922 (2)	0.89897 (15)	0.0761 (9)	
H16A	0.3232	0.2336	0.8967	0.091*	
H16B	0.3619	0.3197	0.9457	0.091*	
C17	0.5135 (4)	0.2754 (2)	0.88228 (13)	0.0788 (10)	
H17A	0.5675	0.3220	0.9060	0.095*	
H17B	0.5398	0.2129	0.8978	0.095*	
C18	0.5657 (2)	0.20575 (15)	0.75882 (10)	0.0402 (5)	
C19	0.6952 (2)	0.22920 (17)	0.71836 (11)	0.0452 (5)	
C20	0.5949 (2)	0.11036 (17)	0.79611 (10)	0.0456 (5)	
C21	0.7328 (2)	0.08641 (17)	0.78283 (11)	0.0482 (6)	
C22	0.7901 (2)	0.15161 (18)	0.73715 (12)	0.0491 (6)	
C23	0.9189 (3)	0.1410 (2)	0.71638 (14)	0.0692 (8)	
H23	0.9576	0.1838	0.6855	0.083*	
C24	0.9880 (3)	0.0645 (3)	0.74306 (16)	0.0816 (9)	
H24	1.0744	0.0563	0.7299	0.098*	
C25	0.9311 (3)	0.0003 (3)	0.78893 (17)	0.0795 (9)	
H25	0.9800	−0.0500	0.8061	0.095*	
C26	0.8033 (3)	0.00942 (19)	0.80953 (14)	0.0639 (7)	
H26	0.7651	−0.0340	0.8401	0.077*	
C27	0.4551 (2)	0.07495 (14)	0.48296 (10)	0.0392 (5)	
C28	0.3612 (2)	0.10843 (16)	0.43596 (11)	0.0434 (5)	
H28	0.3541	0.1732	0.4284	0.052*	
C29	0.2783 (2)	0.04784 (17)	0.40026 (12)	0.0515 (6)	
H29	0.2163	0.0718	0.3692	0.062*	
C30	0.2885 (3)	−0.04835 (18)	0.41119 (14)	0.0614 (7)	
H30	0.2332	−0.0895	0.3873	0.074*	
C31	0.3803 (3)	−0.08373 (18)	0.45731 (14)	0.0678 (8)	
H31	0.3862	−0.1486	0.4648	0.081*	
C32	0.4644 (3)	−0.02266 (16)	0.49279 (12)	0.0546 (6)	
H32	0.5272	−0.0471	0.5232	0.066*	
C33	0.4248 (2)	0.10128 (17)	0.67921 (11)	0.0453 (5)	
N1	0.54457 (16)	0.28914 (12)	0.50512 (8)	0.0385 (4)	
N2	0.5264 (2)	0.28416 (13)	0.80428 (10)	0.0507 (5)	
N3	0.3949 (2)	0.02553 (16)	0.66654 (12)	0.0653 (6)	
O1	0.4773 (2)	0.67002 (12)	0.43580 (11)	0.0735 (6)	
O2	0.67202 (16)	0.21280 (12)	0.41786 (9)	0.0542 (4)	
O3	0.66478 (15)	0.22531 (11)	0.64393 (7)	0.0463 (4)	
O4	0.7423 (2)	0.31898 (13)	0.73731 (10)	0.0601 (5)	
O5	0.51536 (18)	0.06443 (13)	0.82903 (9)	0.0626 (5)	
H4A	0.679 (3)	0.335 (2)	0.7702 (17)	0.076 (10)*	

### Atomic displacement parameters (Å^2^)

**Table:**

	*U*^11^	*U*^22^	*U*^33^	*U*^12^	*U*^13^	*U*^23^
C1	0.151 (4)	0.0565 (19)	0.136 (3)	0.018 (2)	0.072 (3)	0.040 (2)
C2	0.0609 (15)	0.0398 (11)	0.0449 (12)	−0.0010 (11)	0.0029 (11)	0.0046 (9)
C3	0.0504 (14)	0.0484 (13)	0.0483 (12)	−0.0070 (11)	0.0073 (11)	0.0095 (10)
C4	0.0416 (13)	0.0462 (12)	0.0490 (12)	0.0032 (10)	0.0024 (10)	0.0061 (10)
C5	0.0401 (11)	0.0389 (10)	0.0302 (9)	−0.0009 (9)	−0.0017 (9)	0.0016 (8)
C6	0.0495 (14)	0.0436 (12)	0.0453 (11)	−0.0018 (10)	0.0096 (10)	0.0053 (10)
C7	0.0576 (15)	0.0469 (13)	0.0569 (14)	0.0086 (11)	0.0116 (12)	0.0016 (11)
C8	0.0322 (10)	0.0483 (12)	0.0378 (10)	0.0010 (10)	0.0003 (9)	−0.0014 (9)
C9	0.0412 (11)	0.0396 (11)	0.0351 (10)	0.0050 (9)	−0.0026 (9)	0.0026 (8)
C10	0.0394 (11)	0.0368 (10)	0.0327 (9)	−0.0013 (9)	0.0003 (8)	0.0035 (8)
C11	0.0420 (12)	0.0413 (11)	0.0348 (10)	−0.0033 (9)	−0.0035 (9)	0.0030 (8)
C12	0.0450 (12)	0.0366 (11)	0.0336 (9)	−0.0028 (9)	−0.0017 (9)	−0.0003 (8)
C13	0.0480 (14)	0.0573 (14)	0.0406 (11)	0.0020 (11)	0.0004 (10)	−0.0001 (10)
C14	0.0569 (15)	0.0469 (13)	0.0491 (12)	0.0064 (11)	−0.0008 (11)	−0.0037 (10)
C15	0.0767 (19)	0.0723 (18)	0.0634 (16)	0.0013 (16)	0.0074 (14)	−0.0204 (15)
C16	0.114 (3)	0.0599 (17)	0.0547 (15)	−0.0283 (17)	0.0223 (16)	−0.0133 (13)
C17	0.121 (3)	0.075 (2)	0.0403 (13)	0.0251 (19)	−0.0113 (16)	−0.0136 (13)
C18	0.0483 (13)	0.0414 (11)	0.0311 (9)	−0.0035 (10)	−0.0034 (9)	0.0005 (9)
C19	0.0500 (13)	0.0498 (13)	0.0357 (10)	−0.0089 (11)	−0.0074 (10)	0.0028 (9)
C20	0.0567 (15)	0.0481 (13)	0.0319 (10)	−0.0025 (11)	−0.0041 (10)	0.0023 (9)
C21	0.0567 (15)	0.0495 (13)	0.0386 (11)	0.0023 (11)	−0.0093 (10)	−0.0012 (10)
C22	0.0466 (14)	0.0635 (15)	0.0371 (10)	0.0009 (11)	−0.0088 (10)	−0.0039 (10)
C23	0.0532 (17)	0.101 (2)	0.0531 (14)	0.0014 (16)	−0.0042 (13)	0.0038 (15)
C24	0.0574 (18)	0.121 (3)	0.0660 (17)	0.0263 (19)	−0.0088 (15)	−0.0056 (19)
C25	0.076 (2)	0.084 (2)	0.0776 (19)	0.0295 (18)	−0.0236 (17)	−0.0081 (17)
C26	0.077 (2)	0.0542 (15)	0.0610 (15)	0.0083 (14)	−0.0172 (15)	0.0064 (13)
C27	0.0474 (12)	0.0361 (10)	0.0342 (10)	0.0048 (9)	0.0024 (9)	0.0014 (8)
C28	0.0452 (13)	0.0392 (11)	0.0459 (11)	0.0036 (10)	−0.0009 (10)	0.0013 (10)
C29	0.0522 (14)	0.0528 (14)	0.0494 (13)	0.0003 (11)	−0.0089 (11)	0.0015 (11)
C30	0.0813 (19)	0.0471 (14)	0.0558 (14)	−0.0145 (13)	−0.0054 (14)	−0.0070 (11)
C31	0.109 (2)	0.0323 (12)	0.0621 (15)	−0.0002 (14)	−0.0115 (16)	0.0003 (11)
C32	0.0758 (17)	0.0388 (12)	0.0491 (12)	0.0119 (12)	−0.0102 (12)	0.0030 (10)
C33	0.0511 (14)	0.0466 (13)	0.0382 (10)	−0.0049 (10)	−0.0031 (10)	0.0017 (10)
N1	0.0413 (10)	0.0396 (9)	0.0345 (8)	0.0001 (8)	0.0041 (7)	0.0040 (7)
N2	0.0653 (13)	0.0451 (10)	0.0416 (9)	0.0050 (10)	−0.0082 (9)	−0.0075 (8)
N3	0.0829 (17)	0.0497 (13)	0.0633 (13)	−0.0155 (12)	−0.0078 (12)	−0.0038 (11)
O1	0.0946 (15)	0.0422 (9)	0.0838 (12)	0.0107 (10)	0.0267 (12)	0.0173 (9)
O2	0.0499 (10)	0.0567 (10)	0.0560 (10)	−0.0011 (8)	0.0167 (8)	−0.0038 (8)
O3	0.0413 (9)	0.0605 (10)	0.0371 (8)	−0.0055 (7)	−0.0031 (6)	0.0063 (7)
O4	0.0670 (12)	0.0543 (10)	0.0590 (10)	−0.0237 (9)	−0.0130 (10)	0.0045 (9)
O5	0.0707 (12)	0.0609 (11)	0.0563 (9)	−0.0074 (10)	0.0027 (9)	0.0211 (8)

### Geometric parameters (Å, º)

**Table:**

C1—O1	1.434 (4)	C15—H15B	0.9700
C1—H1A	0.9600	C16—C17	1.506 (5)
C1—H1B	0.9600	C16—H16A	0.9700
C1—H1C	0.9600	C16—H16B	0.9700
C2—O1	1.382 (3)	C17—N2	1.483 (3)
C2—C7	1.385 (3)	C17—H17A	0.9700
C2—C3	1.391 (3)	C17—H17B	0.9700
C3—C4	1.388 (3)	C18—N2	1.459 (3)
C3—H3	0.9300	C18—C20	1.550 (3)
C4—C5	1.391 (3)	C18—C19	1.571 (3)
C4—H4	0.9300	C19—O4	1.405 (3)
C5—C6	1.388 (3)	C19—O3	1.440 (2)
C5—N1	1.424 (3)	C19—C22	1.511 (3)
C6—C7	1.385 (3)	C20—O5	1.215 (3)
C6—H6	0.9300	C20—C21	1.480 (4)
C7—H7	0.9300	C21—C22	1.393 (3)
C8—O2	1.213 (2)	C21—C26	1.402 (3)
C8—N1	1.373 (3)	C22—C23	1.390 (4)
C8—C9	1.536 (3)	C23—C24	1.388 (4)
C9—C27	1.509 (3)	C23—H23	0.9300
C9—C10	1.572 (3)	C24—C25	1.385 (5)
C9—H9	0.9800	C24—H24	0.9300
C10—N1	1.495 (2)	C25—C26	1.377 (4)
C10—C11	1.533 (3)	C25—H25	0.9300
C10—H10	0.9800	C26—H26	0.9300
C11—O3	1.439 (3)	C27—C28	1.394 (3)
C11—C12	1.564 (3)	C27—C32	1.396 (3)
C11—H11	0.9800	C28—C29	1.384 (3)
C12—C33	1.484 (3)	C28—H28	0.9300
C12—C13	1.541 (3)	C29—C30	1.380 (3)
C12—C18	1.581 (3)	C29—H29	0.9300
C13—C14	1.532 (3)	C30—C31	1.378 (4)
C13—H13A	0.9700	C30—H30	0.9300
C13—H13B	0.9700	C31—C32	1.394 (4)
C14—N2	1.497 (3)	C31—H31	0.9300
C14—C15	1.535 (4)	C32—H32	0.9300
C14—H14	0.9800	C33—N3	1.140 (3)
C15—C16	1.505 (4)	O4—H4A	0.93 (3)
C15—H15A	0.9700		
			
O1—C1—H1A	109.5	C17—C16—H16A	111.0
O1—C1—H1B	109.5	C15—C16—H16B	111.0
H1A—C1—H1B	109.5	C17—C16—H16B	111.0
O1—C1—H1C	109.5	H16A—C16—H16B	109.0
H1A—C1—H1C	109.5	N2—C17—C16	106.3 (2)
H1B—C1—H1C	109.5	N2—C17—H17A	110.5
O1—C2—C7	116.5 (2)	C16—C17—H17A	110.5
O1—C2—C3	123.8 (2)	N2—C17—H17B	110.5
C7—C2—C3	119.6 (2)	C16—C17—H17B	110.5
C4—C3—C2	119.8 (2)	H17A—C17—H17B	108.7
C4—C3—H3	120.1	N2—C18—C20	116.61 (16)
C2—C3—H3	120.1	N2—C18—C19	111.12 (18)
C3—C4—C5	120.2 (2)	C20—C18—C19	103.89 (18)
C3—C4—H4	119.9	N2—C18—C12	105.82 (17)
C5—C4—H4	119.9	C20—C18—C12	114.56 (17)
C6—C5—C4	119.87 (19)	C19—C18—C12	104.23 (15)
C6—C5—N1	120.51 (18)	O4—C19—O3	110.96 (17)
C4—C5—N1	119.61 (18)	O4—C19—C22	111.93 (19)
C7—C6—C5	119.7 (2)	O3—C19—C22	109.98 (19)
C7—C6—H6	120.2	O4—C19—C18	111.07 (19)
C5—C6—H6	120.2	O3—C19—C18	106.35 (17)
C6—C7—C2	120.7 (2)	C22—C19—C18	106.31 (18)
C6—C7—H7	119.6	O5—C20—C21	127.6 (2)
C2—C7—H7	119.6	O5—C20—C18	124.5 (2)
O2—C8—N1	132.2 (2)	C21—C20—C18	107.93 (19)
O2—C8—C9	135.0 (2)	C22—C21—C26	121.1 (2)
N1—C8—C9	92.72 (15)	C22—C21—C20	111.0 (2)
C27—C9—C8	112.06 (16)	C26—C21—C20	127.8 (2)
C27—C9—C10	117.04 (18)	C23—C22—C21	120.4 (2)
C8—C9—C10	85.62 (15)	C23—C22—C19	128.9 (2)
C27—C9—H9	113.1	C21—C22—C19	110.6 (2)
C8—C9—H9	113.1	C24—C23—C22	118.1 (3)
C10—C9—H9	113.1	C24—C23—H23	121.0
N1—C10—C11	113.07 (16)	C22—C23—H23	121.0
N1—C10—C9	86.83 (14)	C25—C24—C23	121.4 (3)
C11—C10—C9	118.45 (17)	C25—C24—H24	119.3
N1—C10—H10	112.1	C23—C24—H24	119.3
C11—C10—H10	112.1	C26—C25—C24	121.2 (3)
C9—C10—H10	112.1	C26—C25—H25	119.4
O3—C11—C10	110.40 (17)	C24—C25—H25	119.4
O3—C11—C12	104.37 (16)	C25—C26—C21	117.7 (3)
C10—C11—C12	117.12 (17)	C25—C26—H26	121.1
O3—C11—H11	108.2	C21—C26—H26	121.1
C10—C11—H11	108.2	C28—C27—C32	117.9 (2)
C12—C11—H11	108.2	C28—C27—C9	121.37 (18)
C33—C12—C13	111.82 (19)	C32—C27—C9	120.69 (19)
C33—C12—C11	111.20 (17)	C29—C28—C27	121.8 (2)
C13—C12—C11	114.83 (18)	C29—C28—H28	119.1
C33—C12—C18	113.06 (18)	C27—C28—H28	119.1
C13—C12—C18	103.73 (16)	C30—C29—C28	119.4 (2)
C11—C12—C18	101.59 (16)	C30—C29—H29	120.3
C14—C13—C12	103.85 (19)	C28—C29—H29	120.3
C14—C13—H13A	111.0	C31—C30—C29	120.3 (2)
C12—C13—H13A	111.0	C31—C30—H30	119.9
C14—C13—H13B	111.0	C29—C30—H30	119.9
C12—C13—H13B	111.0	C30—C31—C32	120.3 (2)
H13A—C13—H13B	109.0	C30—C31—H31	119.9
N2—C14—C13	104.49 (18)	C32—C31—H31	119.9
N2—C14—C15	105.2 (2)	C31—C32—C27	120.4 (2)
C13—C14—C15	116.4 (2)	C31—C32—H32	119.8
N2—C14—H14	110.1	C27—C32—H32	119.8
C13—C14—H14	110.1	N3—C33—C12	177.5 (3)
C15—C14—H14	110.1	C8—N1—C5	131.91 (16)
C16—C15—C14	104.3 (2)	C8—N1—C10	94.77 (16)
C16—C15—H15A	110.9	C5—N1—C10	132.56 (16)
C14—C15—H15A	110.9	C18—N2—C17	123.04 (19)
C16—C15—H15B	110.9	C18—N2—C14	110.58 (16)
C14—C15—H15B	110.9	C17—N2—C14	108.1 (2)
H15A—C15—H15B	108.9	C2—O1—C1	116.7 (2)
C15—C16—C17	103.8 (2)	C11—O3—C19	107.76 (16)
C15—C16—H16A	111.0	C19—O4—H4A	98 (2)
			
O1—C2—C3—C4	179.2 (2)	O5—C20—C21—C26	−5.0 (4)
C7—C2—C3—C4	−0.2 (4)	C18—C20—C21—C26	176.2 (2)
C2—C3—C4—C5	0.2 (4)	C26—C21—C22—C23	0.7 (4)
C3—C4—C5—C6	0.4 (3)	C20—C21—C22—C23	−178.5 (2)
C3—C4—C5—N1	179.5 (2)	C26—C21—C22—C19	−178.0 (2)
C4—C5—C6—C7	−1.1 (3)	C20—C21—C22—C19	2.8 (3)
N1—C5—C6—C7	179.9 (2)	O4—C19—C22—C23	−56.9 (3)
C5—C6—C7—C2	1.1 (4)	O3—C19—C22—C23	66.9 (3)
O1—C2—C7—C6	−179.8 (2)	C18—C19—C22—C23	−178.4 (2)
C3—C2—C7—C6	−0.5 (4)	O4—C19—C22—C21	121.6 (2)
O2—C8—C9—C27	−60.9 (3)	O3—C19—C22—C21	−114.6 (2)
N1—C8—C9—C27	115.56 (18)	C18—C19—C22—C21	0.2 (2)
O2—C8—C9—C10	−178.4 (3)	C21—C22—C23—C24	−0.7 (4)
N1—C8—C9—C10	−1.89 (15)	C19—C22—C23—C24	177.7 (2)
C27—C9—C10—N1	−110.83 (17)	C22—C23—C24—C25	0.2 (4)
C8—C9—C10—N1	1.73 (14)	C23—C24—C25—C26	0.4 (5)
C27—C9—C10—C11	134.57 (19)	C24—C25—C26—C21	−0.4 (4)
C8—C9—C10—C11	−112.87 (18)	C22—C21—C26—C25	−0.2 (4)
N1—C10—C11—O3	−68.3 (2)	C20—C21—C26—C25	178.9 (2)
C9—C10—C11—O3	31.1 (2)	C8—C9—C27—C28	−46.0 (3)
N1—C10—C11—C12	172.52 (17)	C10—C9—C27—C28	50.6 (3)
C9—C10—C11—C12	−88.1 (2)	C8—C9—C27—C32	132.5 (2)
O3—C11—C12—C33	−85.7 (2)	C10—C9—C27—C32	−130.9 (2)
C10—C11—C12—C33	36.7 (3)	C32—C27—C28—C29	0.6 (3)
O3—C11—C12—C13	146.07 (18)	C9—C27—C28—C29	179.1 (2)
C10—C11—C12—C13	−91.5 (2)	C27—C28—C29—C30	−0.1 (4)
O3—C11—C12—C18	34.9 (2)	C28—C29—C30—C31	0.1 (4)
C10—C11—C12—C18	157.24 (18)	C29—C30—C31—C32	−0.6 (4)
C33—C12—C13—C14	154.06 (18)	C30—C31—C32—C27	1.1 (4)
C11—C12—C13—C14	−78.0 (2)	C28—C27—C32—C31	−1.1 (4)
C18—C12—C13—C14	31.9 (2)	C9—C27—C32—C31	−179.6 (2)
C12—C13—C14—N2	−33.1 (2)	O2—C8—N1—C5	7.9 (4)
C12—C13—C14—C15	−148.5 (2)	C9—C8—N1—C5	−168.7 (2)
N2—C14—C15—C16	−26.4 (3)	O2—C8—N1—C10	178.6 (2)
C13—C14—C15—C16	88.7 (3)	C9—C8—N1—C10	1.98 (16)
C14—C15—C16—C17	35.1 (3)	C6—C5—N1—C8	153.4 (2)
C15—C16—C17—N2	−31.0 (3)	C4—C5—N1—C8	−25.6 (3)
C33—C12—C18—N2	−140.51 (18)	C6—C5—N1—C10	−14.0 (3)
C13—C12—C18—N2	−19.2 (2)	C4—C5—N1—C10	167.0 (2)
C11—C12—C18—N2	100.25 (19)	C11—C10—N1—C8	117.73 (19)
C33—C12—C18—C20	−10.6 (3)	C9—C10—N1—C8	−1.94 (15)
C13—C12—C18—C20	110.7 (2)	C11—C10—N1—C5	−71.6 (3)
C11—C12—C18—C20	−129.86 (18)	C9—C10—N1—C5	168.7 (2)
C33—C12—C18—C19	102.2 (2)	C20—C18—N2—C17	−0.3 (3)
C13—C12—C18—C19	−136.46 (18)	C19—C18—N2—C17	−119.0 (3)
C11—C12—C18—C19	−17.0 (2)	C12—C18—N2—C17	128.4 (2)
N2—C18—C19—O4	1.3 (2)	C20—C18—N2—C14	−130.1 (2)
C20—C18—C19—O4	−124.83 (18)	C19—C18—N2—C14	111.1 (2)
C12—C18—C19—O4	114.88 (18)	C12—C18—N2—C14	−1.5 (2)
N2—C18—C19—O3	−119.50 (18)	C16—C17—N2—C18	−116.3 (3)
C20—C18—C19—O3	114.33 (18)	C16—C17—N2—C14	14.6 (3)
C12—C18—C19—O3	−6.0 (2)	C13—C14—N2—C18	21.7 (2)
N2—C18—C19—C22	123.32 (19)	C15—C14—N2—C18	144.8 (2)
C20—C18—C19—C22	−2.8 (2)	C13—C14—N2—C17	−115.7 (2)
C12—C18—C19—C22	−123.13 (17)	C15—C14—N2—C17	7.4 (3)
N2—C18—C20—O5	63.0 (3)	C7—C2—O1—C1	−174.6 (3)
C19—C18—C20—O5	−174.4 (2)	C3—C2—O1—C1	6.1 (4)
C12—C18—C20—O5	−61.4 (3)	C10—C11—O3—C19	−167.85 (16)
N2—C18—C20—C21	−118.1 (2)	C12—C11—O3—C19	−41.2 (2)
C19—C18—C20—C21	4.5 (2)	O4—C19—O3—C11	−91.4 (2)
C12—C18—C20—C21	117.51 (19)	C22—C19—O3—C11	144.18 (19)
O5—C20—C21—C22	174.1 (2)	C18—C19—O3—C11	29.5 (2)
C18—C20—C21—C22	−4.7 (2)		

### Hydrogen-bond geometry (Å, º)

**Table:**

*D*—H···*A*	*D*—H	H···*A*	*D*···*A*	*D*—H···*A*
C10—H10···O2^i^	0.98	2.43	3.337 (3)	154
C13—H13*A*···O2^i^	0.97	2.41	3.321 (3)	156
C29—H29···O4^i^	0.93	2.55	3.228 (3)	130
O4—H4*A*···N2	0.93 (3)	1.84 (3)	2.602 (3)	137 (3)

Symmetry code: (i) *x*−1/2, −*y*+1/2, −*z*+1.
